# Supplementary material for: Analysis of the Anticipatory Behavior Formation Mechanism Induced by Methamphetamine Using a Single Hair
Source: Cells. 2023 Feb 17;12(4):654. doi: 10.3390/cells12040654 (PMC9954696; doi:10.3390/cells12040654)
Supplement: Supplementary file 1 [file cells-12-00654-s001.zip › cells-2002797-supplementary.pdf]

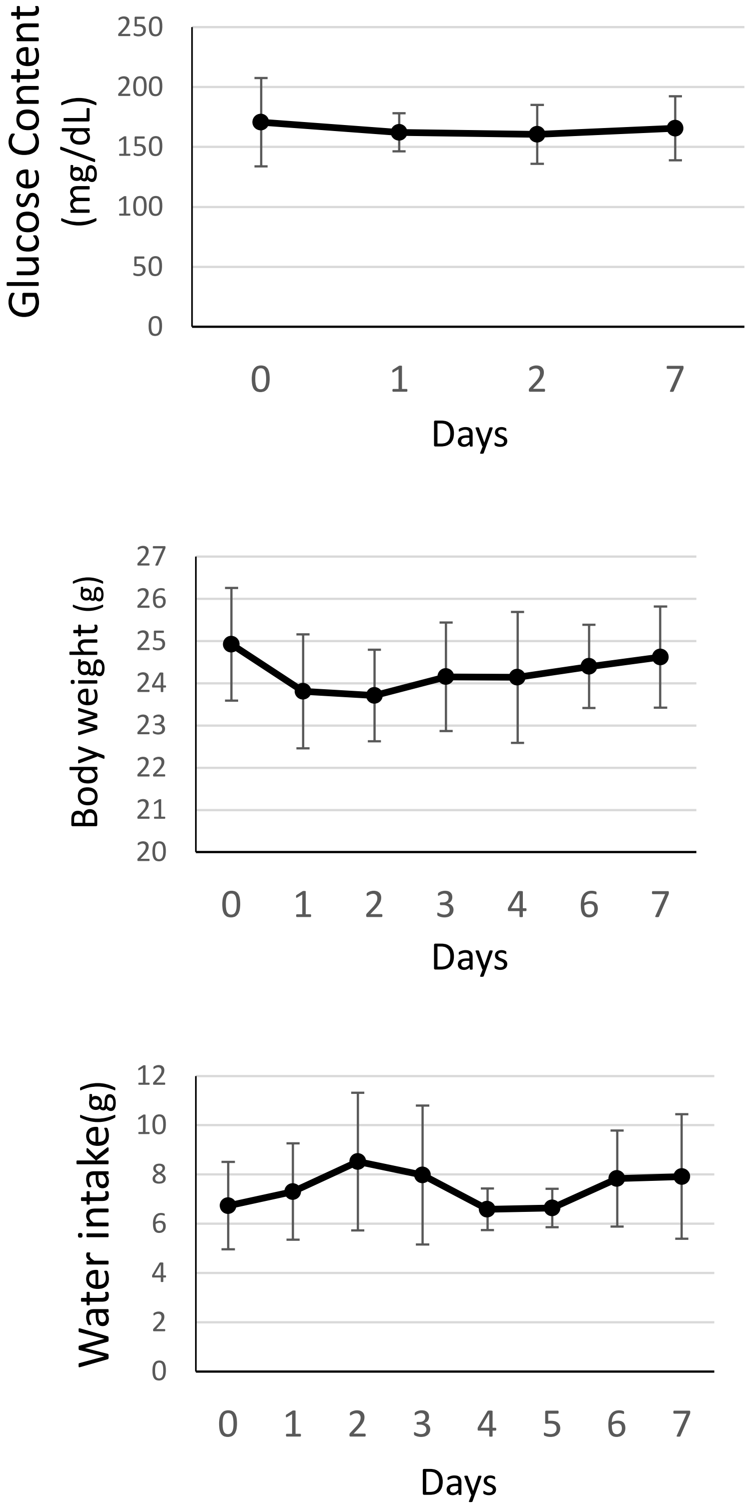


**Supplementary Figure S1. Effects of MAP on blood glucose, body weight, and water intake:**

Blood glucose content, body weight and water intake during MAP injection. Each plot indicates the mean ± S.D. (*n*=4). There were no differences in blood glucose, body weight, and water intake during MAP injection.

Supplementary FigureS1

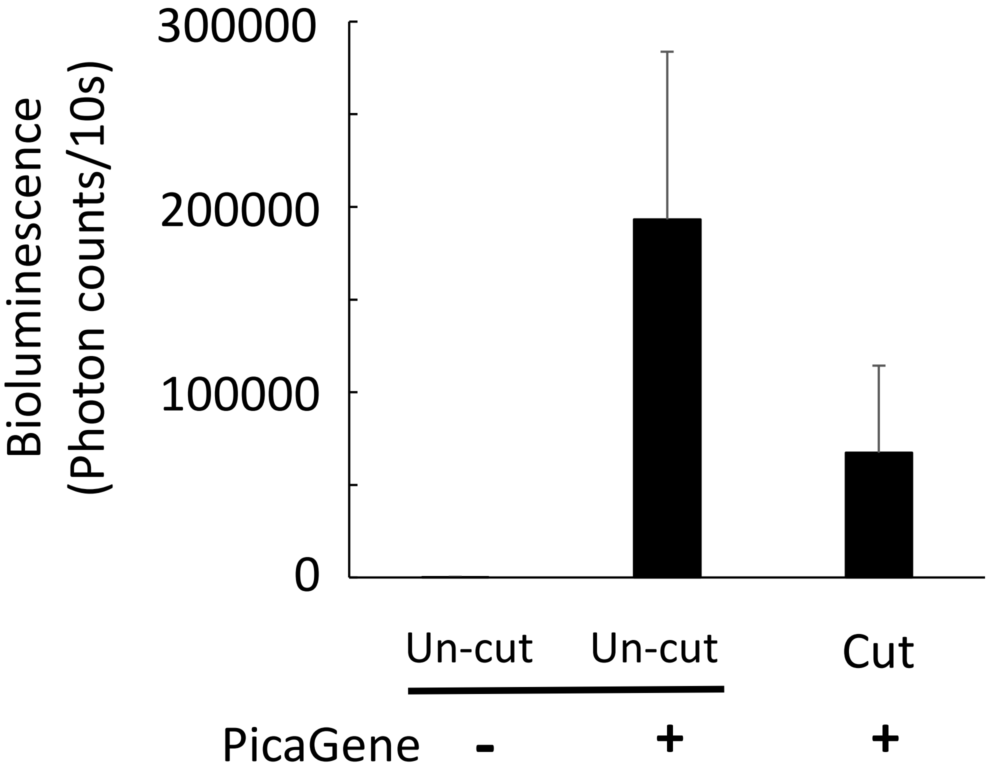


**Supplementary Figure** S2. ***Per1*****expression in a single whisker hair using the direct method:**

– and + indicate the measurement of *Per1* expression with no PicaGene solution and with PicaGene solution, respectively. The un-cut group indicates direct measurement of *Per1* expression in a whisker hair. The cut group indicates the measurement of *Per1* expression from longitudinally cut whisker samples (vertically halved samples). Statistical significance was determined by one-way ANOVA followed by Dunnett's test ($n$=4 animals) (*P<0.05 vs un-cut (-) group).

Supplementary FigureS2
